# Chip-Sized Lensless Holographic Microscope for Real-Time On-Chip Biological Sensing

**DOI:** 10.3390/s25175247

**Published:** 2025-08-23

**Authors:** Sofía Moncada-Madrazo, Sergio Moreno, Oriol Caravaca, Joan Canals, Natalia Castro, Manel López, Javier Ramón-Azcón, Anna Vilà, Ángel Diéguez

**Affiliations:** 1 Department of Electronic and Biomedical Engineering, Universitat de Barcelona, Martí i Franquès 1, 08028 Barcelona, Spain; sergiomoreno@ub.edu (S.M.); oriolcaravaca@ub.edu (O.C.); canals@ub.edu (J.C.); manel.lopez@ub.edu (M.L.); anna.vila@ub.edu (A.V.); 2 Institute of Nanoscience and Nanotechnology (INUB), Universitat de Barcelona, 08028 Barcelona, Spain; 3 Institute for Bioengineering of Catalonia (IBEC), The Barcelona Institute of Science and Technology (BIST), 08028 Barcelona, Spain; ncastro2@ibecbarcelona.eu (N.C.); jramon@ibecbarcelona.eu (J.R.-A.)

**Keywords:** compact microscope, lensless, holography, lab-on-a-chip, real-time monitoring, angiogenesis, fermentation, zebrafish

## Abstract

Microscopy is a fundamental tool in biological research. However, conventional microscopes require manual operation and depend on user and equipment availability, limiting their suitability for continuous observation. Moreover, their size and complexity make them impractical for in situ experimentation. In this work, we present a novel, compact, affordable, and portable microscope that enables continuous in situ monitoring by being placed directly on biological samples. This chip-sized lensless holographic microscope (CLHM) is specifically designed to overcome the limitations of traditional microscopy. The device consists solely of an ultra-compact, state-of-the-art micro-LED display and a CMOS sensor, all enclosed within a 3D-printed housing. This unique light source enables a size that is markedly smaller than any comparable technology, allowing a resolution of 2.19 μm within a 7 mm distance between the light source and the camera. This paper demonstrates the CLHM’s versatility by monitoring in vitro models and performing whole-organism morphological analyses of small specimens. These experiments underscore its potential as an on-platform sensing device for continuous, in situ biological monitoring across diverse models.

## 1. Introduction

The growing demand for drug development and disease modeling has driven the implementation of diverse in vitro and in vivo systems. In vitro platforms include 2D and 3D cellular models, organoids, microfluidic organs-on-a-chip (OOC), and microfluidic single-cell interaction analysis platforms [1]. In vivo models commonly involve rodents, zebrafish, and other larger species. Although these systems would largely benefit from continuous monitoring and analysis, most conventional microscopes are bulky, expensive, non-portable, and not easily scalable, hindering widespread access, particularly under budget constraints, reducing the number of systems that can be monitored simultaneously, and complicating in situ experimentation.

Such challenges can be mitigated through alternative imaging approaches, including miniaturized, smartphone-based, and lensless microscopy techniques, which offer greater flexibility, affordability, and integration potential for modern biological research.

### 1.1. Miniaturized Microscopy

Miniaturized microscopes keep the same components of conventional microscopes, such as filters, detectors, lenses, and light sources (i.e., LED), but they are engineered to be compact and lightweight. These devices can be categorized based on their imaging modalities: brightfield, fluorescence, and hybrid systems, among other.

Brightfield miniature microscopy typically incorporates an LED as light source, an optical focusing system, and a CMOS (Complementary Metal-Oxide Semiconductor) camera for image acquisition [2]. Fluorescence miniature microscopes utilize additional optical elements, such as excitation and emission filters and specialized lenses, to detect fluorescent signals from the labeled samples. Common components include an LED light source, optical filters, GRIN (Gradient-Index) lenses, various types of focusing lenses (e.g., liquid, diffuser, objective), and CMOS detectors [3,4,5]. Hybrid miniature modalities combine two or more imaging techniques. Examples include systems integrating both fluorescence and oblique backscattered illumination [6], and fluorescence and brightfield imaging [7].

The design of miniature microscopes enables significant size reduction, making them compact and portable. These advantages are not typically found in conventional microscopes. However, they still have certain limitations. The inclusion of multiple components and the complexity of their arrangement [2,3,4,5,6,7] can increase the risk of misalignment or failure due to mechanical stress or vibrations. Also, the use of GRIN lenses in fluorescence miniature microscopes [3,4,5,6] has limited performance due to out-of-focus fluorescence, as emitted light from regions outside the focal place increases background noise and hinders the resolution of fine details. Additionally, the poor optical sectioning associated with GRIN lenses prevents accurate isolation of in-focus structures [8].

### 1.2. Smartphone-Based Microscopy

Smartphone-based microscopes generally follow two design approaches: one uses a single lens paired with the smartphone [8,9,10], while the other incorporates multiple optical elements, such as excitation and emission filters, dichroic mirrors, and collimating lenses [11,12]. These microscopes support a range of imaging modalities, including brightfield, which often utilizes the smartphone’s built-in flashlight [9,10,11], fluorescence, which requires additional light sources (e.g., LEDs) [13], and hybrid modalities [11].

These microscopes effectively address the size and portability limitations of conventional approaches, but each design has its own drawbacks. Systems with minimal optical elements may suffer from optical aberrations, such as distortions, especially those using ball lenses [8]. On the other hand, smartphone-based microscopes with multiple optical elements [11] demand precise alignment and mechanical stability. Any misalignment or instability can result in blurred, distorted, or inconsistent images.

### 1.3. Lensless Microscopy

In lensless microscopy, lenses are omitted entirely, significantly reducing the overall size of the microscope. A widely adopted implementation is on-chip lensless microscopy [14,15,16], which typically uses a coherent (laser) or partially coherent (LED) light source along with a CCD or CMOS sensor. In this configuration, the sample is usually placed on top of the sensor. Hence the term *on-chip microscopy*. As light passes through the sample, the image sensor captures an interference pattern, or hologram. This hologram encodes both the amplitude and phase information of the transmitted light. The original wavefront produced by the object is digitally reconstructed by using computational backpropagation techniques, allowing visualization of the sample without traditional optics.

Despite its advantages in compactness and simplicity, on-chip microscopy presents several challenges, particularly related to the illumination source. When using laser diodes [14], speckle noise, caused by coherence interference, can degrade image quality. While optical elements like focusing lenses may reduce this effect, they also introduce added complexity and susceptibility to mechanical misalignment. Moreover, laser diodes require precise thermal and power regulation to avoid damaging biological samples, and their narrow spectral bandwidth can lead to chromatic aberrations, limiting their use in color-sensitive applications. Alternatively, LEDs provide broader spectral output and greater compatibility with color-based imaging but have limitations of their own. On-chip microscopes that use LEDs combined with pinholes [15,16,17,18] may suffer from lower spatial resolution, due to reduced coherence and light throughput. Additionally, setups that involve rotating mechanisms to reposition the illumination source [16] can introduce mechanical vibrations, which in turn compromise image stability and quality.

In addition to the on-chip microscopes mentioned above, some lensless microscopes employ shifting or structured illumination. Zheng et al. [19] used a LED array to capture multiple low-resolution images at different angles of illumination and combine them computationally to obtain a higher-resolution image reconstruction. This approach, termed Fourier Ptychographic Microscopy (FPM), specifically uses a low-NA objective and an iterative phase-retrieval procedure in the Fourier domain to recover a high-resolution complex image from many angle-varied frames. Similar concepts are found in other systems where partially coherent light is combined with shifting mechanisms such as a movable mask, diffuser or modulator [20,21,22]. However, these moving optical elements introduce mechanical complexity, slow image acquisition, and make the system more sensitive to misalignment and vibrations. As a result, although some designs may reduce the need for precise positioning, systems that rely on scanning diffusers or masks still face limitations due to stage accuracy and acquisition speed. Meanwhile, motion-free implementations avoid mechanical complexity but require longer multi-frame acquisitions and stricter control over illumination.

As a step ahead to overcome all these limitations, in this article we present a Chip-sized Lensless Holographic Microscope (CLHM), a device that falls into the on-chip lensless microscopy category and uses a compact LED microdisplay array (640 × 480 px) to shift illumination without any movable parts, objectives, or mechanical scanning. Importantly, CLHM employs backpropagation with iterative twin-image suppression, which reduces computational burden of ptychography-based reconstructions for portable in situ use. We describe its working principle, highlight its differences from other on-chip microscopes, and evaluate its experimental resolution. The CLHM’s compact size (6 × 6 × 4.5 cm^3^), cost-effectiveness for large-scale deployment, and compatibility with in situ imaging make it well-suited for a wide range of biological and biomedical applications. Unlike miniature microscopes and smartphone-based systems, it does not rely on components such as lenses, filters, or smartphone cameras, limiting its cost and complexity. The CLHM offers an affordable alternative, particularly valuable for budget-constrained research. Its continuous monitoring of dynamic cellular processes without bulky or expensive equipment enables its use in laboratory and field settings. To demonstrate the versatility of the CLHM, we selected three representative use cases spanning microbial fermentation, vertebrate model organisms, and in vitro tissue culture models. These examples illustrate the microscope’s applicability across multiple spatial and biological scales, from unicellular systems to complex networks.

One key application of the CLHM includes cell monitoring and counting, as a crucial step towards drug validation, immune and cancer therapy, cell invasion, migration and physiology studies, etc. As a particular example of this field, the fermentation process in wine production has been chosen. Continuous observation during fermentation is essential to ensure wine quality by controlling characteristics such as flavor and aroma, preventing contamination, and optimizing fermentation parameters. Monitoring yeast concentration is critical for all types of wine, but especially for sparkling wines, where specific cell densities are required to achieve proper carbonation and maintain consistent quality [23]. By validating CLHM on yeast cells in fermentation, we not only ensure wine quality control but also establish a transferable framework for counting, viability, and physiology monitoring in mammalian systems used in drug discovery and immuno-oncology.

Zebrafish (Danio rerio) is another important model organism supported by the CLHM. It is extensively used in studies related to metabolic disorders (e.g., obesity and diabetes) [24,25], viral infections [26,27], vaccine testing [28], and neurological and development disorders [29]. Its utility stems from its genetic similarity to humans (approximately 70% of the human genes have their counterpart in zebrafish), optical transparency (facilitating real-time in vivo imaging of tissues and cellular processes), and practical advantages such as high fecundity (300 to 600 by a single female at one time), rapid organ development, and low maintenance costs compared to rodents [25]. Additionally, zebrafish can be studied at different stages of development, from the embryo (48–72 h post fertilization), larval, juvenile (4 to 12 weeks post-fertilization), or adult stage, depending on the research needs [30].

As a completely different application, CLHM is also suited for the observation of cultured cells in various configurations. Cellular culture is a laboratory process that involves the growth and proliferation of animal or human cells under specific conditions. In vitro models include: (i) primary cells directly isolated from human or animal tissue [31], (ii) immortalized cell lines, which proliferate indefinitely, and can be cultured through several generations [32], (iii) stem-cells, including embryonic stem cells (hESCs) and induced pluripotent stem cells (hiPSCs)—the latter being reprogrammed somatic cells that retain pluripotency and self-renewal properties [33]. These cells can be cultured in 2D monolayers, 3D extracellular matrices, or organoid systems, and are often co-cultured to study intercellular interactions such as migration, differentiation, viability, and proliferation [34].

In this context, angiogenesis models represent a significant application area for CLHM, particularly as a representative use case among many dynamic biological processes that benefit from continuous, high-resolution imaging. Angiogenesis is a process in which new blood vessels are generated from existing vessels and requires complex molecular and cellular interactions [35]. It was selected for demonstration due to its relevance in biomedical research, the availability of well-established in vitro models, and the dynamic nature of the underlying cellular mechanisms. Researchers commonly employ 2D or 3D culture systems to study angiogenesis in contexts such as cancer therapy and retinal vascular diseases [36]. A widely used model involves human umbilical vein endothelial cells (HUVECs), which are isolated from discarded umbilical cords through non-invasive protocols. HUVECs are preferred due to their accessibility in large quantities and the extensive literature available, facilitating reproducibility and comparison across studies [37].

Beyond these initial demonstrations, the CLHM holds promise for broader applications, such as environmental monitoring, point-of-care diagnostics, and education in low-resource settings. Its scalability and simplicity make it a flexible platform for integration with automated image processing and remote data collection systems, paving the way for new use cases in decentralized and continuous biological analysis.

Before detailing our experimental approach, we note the intended scope of this work. In this study, we present qualitative proof of concept demonstrating CLHM’s capabilities for in situ biological monitoring (e.g., yeast cells, zebrafish anatomy, angiogenic structures). A comprehensive quantitative evaluation encompassing techniques such as counting vascular junctions in angiogenesis assays, measuring morphological features in zebrafish embryos, and assessing pharmacological effects is beyond the scope of this manuscript and will be addressed in future studies with proper statistical power.

## 2. Materials and Methods

### 2.1. Chip-Sized Microscope (CLHM) Hardware

The CLHM is composed of a CMOS camera DMM 37UX178-ML (from Imaging Source, Bremen, Germany; distributed by IberOptics, Madrid, Spain) and an LED array as a light source μdisplay with 640 × 480 MQW InGaN/GaN LEDs of 1-μm diameter and 4-μm pitch with a display area of 2.64 mm × 2.02 mm (from Jade Bird Display, Shanghai, China).

As in other lensless holographic microscopes [14,15,16], our CLHM uses a partially coherent light source to illuminate the sample and the CMOS sensor captures the hologram. This hologram is then computationally reconstructed using a numerical backpropagation algorithm to retrieve the final sample image.

In contrast to other lensless holographic microscope designs reported in the literature, the CLHM uses a commercial microLED display as its light source near to the sample. The microdisplay employed in our CLHM represents the state of the art in commercially available LED sizes. Each LED in the display has a pitch of 4 μm, offering significantly small emitting area.

Zheng et al. [19] have demonstrated that the adverse effect of limited spatial coherence is reduced as the source diameter (Δs) decreases, in other words, the spatial coherence is improved as the emitting area is reduced. Consequently, using the microLED display provides higher spatial coherence than traditional systems that rely on conventional LEDs with pinholes ranging from 90 to 150 μm [15,16,17,18], as the emitting diameter is significantly smaller. Having a high spatial coherence is critical for high-quality holographic image reconstruction. The improved coherence also enables a shorter distance between the sample and the sensor to a few millimeters (≤10 mm), resulting in a more compact and integrated design than standard LHM setups. Thus, the CLHM achieves high spatial coherence and a compact system design by using a commercially available microdisplay featuring state-of-the-art LED miniaturization.

**Figure 1:**
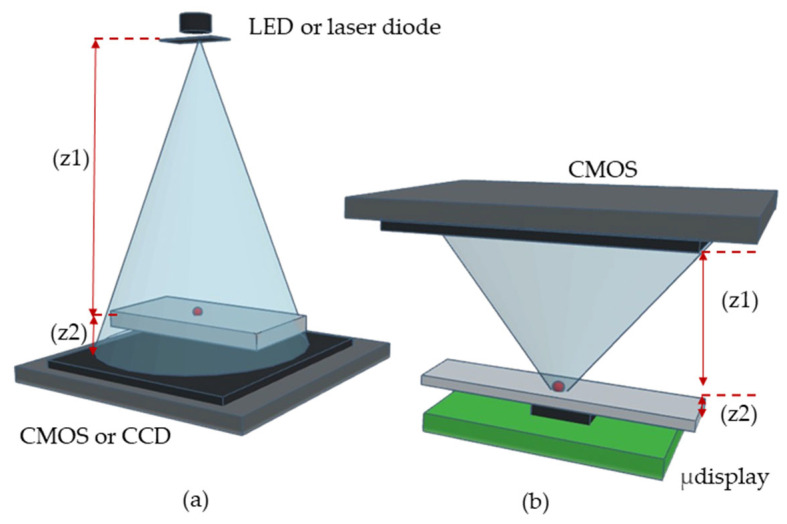
Schematics of (**a**) Lensless-holographic-microscope conventional setup in which the sample is close to the detector (CMOS or CCD). In this configuration, z1 represents the distance between the light source and the sample z2 is the distance between the sample and the CMOS. (**b**) CLHM setup in which the sample is close to the light source (microdisplay). In this case, z1 denotes the distance between the sample and CMOS and the distance z2 corresponds to the approximate distance between the light source and the sample.

**Figure 2:**
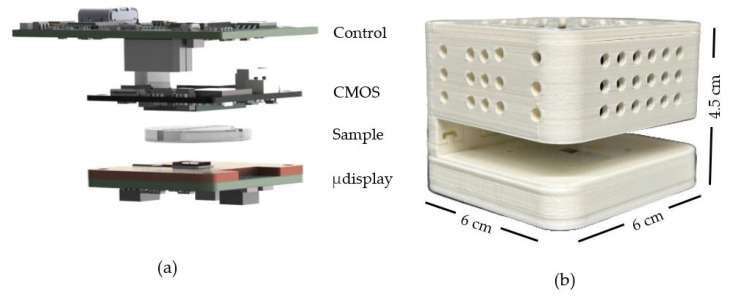
(**a**) Exploded components, i.e., CMOS camera with its control board, sample and microdisplay with its own control board, and (**b**) external appearance of our CLHM in a 3D-printed case.

To illustrate our approach, Figure 1 compares the CLHM configuration with conventional setups. In the traditional configuration (Figure 1a), the sample is placed close to the detector (z2 = 1–5 mm), while the light source is located at a larger distance (z1 = 50–100 mm). In contrast, the CLHM configuration (Figure 1b) places the sample much closer to the light source (z2 = 100–500 μm), allowing a significant reduction in the light source-to-sample distance (z1) to only a few millimeters. Placing the sample away from the sensor can avoid potential sample-heating issues, as the image sensor is typically the component that generates the most heat during operation. This makes the CLHM more suitable for biological research. Figure 2 translates the conceptual idea from Figure 1b into a physical implementation, showcasing the stacked components, including the control board, CMOS detector, sample, microdisplay, and the 3D housing that encases these components.

### 2.2. Chip-Sized Microscope (CLHM) Software

The CLHM’s software (unxip-0.5.7) application was developed in Python (3.9) using a Qt-based graphical interface to guide users through a structured imaging and reconstruction pipeline that produces the final image. The workflow consists of the following stages:

(i) Parameter configuration: Users first define camera settings—such as frame rate, pixel pitch, gain, and exposure time—as well as display parameters, including LED pitch, emission wavelength, intensity, luminance, and current bias. The desired LED grid size (e.g., 5 × 5) is also specified.
(ii) Hologram acquisition: A series of images is captured as the LED array sequentially illuminates the sample. For example, a 5 × 5 grid results in 25 individual holographic segments.
(iii) Normalization: Each image is corrected for LED intensity variations and ambient light effects via background subtraction. This step homogenizes the illumination across all segments.
(iv) Stitching: The normalized segments are spatially combined to form a single composite hologram that represents the full field of view.
(v) Holographic reconstruction: A numerical backpropagation algorithm refocuses the wavefront to the sample plane, and twin-image correction is applied to reduce reconstruction artifacts.

The mathematical formulation of this process was detailed in previous work [21]. Finally, to reduce twin-image artifacts, a common issue in in-line holography, an iterative phase retrieval algorithm is used. This method refines the amplitude and phase information over successive iterations, improving image clarity and resolution.

### 2.3. Experimental Setups

#### 2.3.1. CLHM-Resolution Analysis Setup

To evaluate the image resolution of the CLHM, a USAF 1951 Positive Resolution test target R1L1S1P (from Thorlabs, Bergkirchen, Germany) was used. This target consists of a patterned glass substrate (18 × 18 × 1.5 mm) featuring standardized groups of lines with widths and spacings ranging from 125 µm down to 2.19 µm. The experimental setup included the CLHM, a compact precision Z stage L-306, (from Physik Instrumente, Karlsruhe, Germany) positioner, a stepper motor controller C-663.12 Mercury (from Physik Instrumente, Karlsruhe, Germany), and a 3D home-made sample holder that was fixed to the compact precision Z stage.

Initially, the CMOS camera was positioned 11 mm above the sample (position P1). The μdisplay was aligned with the Z-stage, and the resolution target was mounted onto the 3D-printed holder. An 8 × 8 LED grid was activated sequentially to capture the hologram, resulting in 64 individual holographic images. The selected step size was 10 LEDs, corresponding to a physical pitch of 4 μm per LED. These parameters ensured sufficient overlap between images to allow accurate image stitching and high-quality image reconstruction. These images were normalized, stitched into a composite, digitally backpropagated to the sample plane, and subjected to twin-image correction. This procedure was repeated across five vertical positions, each separated by a 1 mm decrement in the distance between the CMOS sensor and the sample, ranging from 11 mm (P1) to 7 mm (P5). At each position, the same acquisition and reconstruction process was performed.

#### 2.3.2. Microfluidic Setup for Monitoring Wine Samples

**Figure 3:**
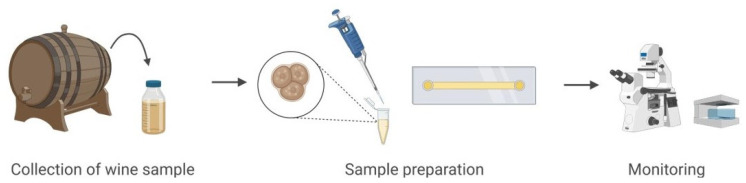
Wine sample preparation and setup.

To evaluate the CLHM’s ability to monitor unicellular specimens, we analyzed yeast activity during wine fermentation. The sample was collected under sterile conditions and immediately transferred to a single-channel μ-Slide (IBIDI, 80167). A volume of 50 µL of the wine sample was carefully pipetted into the slide, ensuring proper sample distribution and adherence to the imaging plane. Figure 3 illustrates the sample preparation workflow, from collection to mounting and imaging with the CLHM. This configuration enabled real-time observation of yeast cells and microstructures within the wine, demonstrating the microscope’s capability for in situ monitoring of fermentation processes.

#### 2.3.3. Zebrafish Imaging Setup

**Figure 4:**
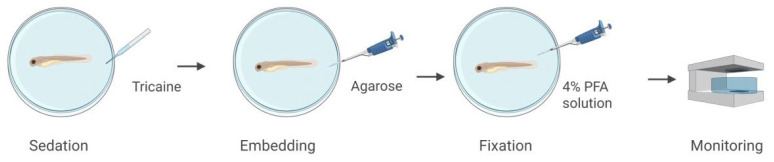
Zebrafish sample preparation and setup.

To demonstrate the CLHM’s ability to capture anatomical features in vertebrate models, we imaged a 72 h post-fertilization (hpf) zebrafish embryo. The specimen was sedated with tricaine, immobilized in 1% low-melting-point agarose in a 35 mm MatTek dish with a No. 1.5, 14 mm coverslip, and subsequently fixed with 4% paraformaldehyde (PFA) (Figure 4).

To capture the full body of the larva, multiple image segments were acquired and later combined into a composite image. For the first region, targeting the head and the upper body, a 5 × 5 LED grid was used to ensure high detail and inclusion of all relevant anatomical features. Subsequent sections of the body were captured using 4 × 4 LED grids, allowing for efficient coverage while maintaining resolution. Each grid was processed through the abovementioned pipeline: normalization, stitching, backpropagation, and twin image removal to obtain a high-resolution image of the entire zebrafish.

#### 2.3.4. Angiogenesis Assay for Tissue and Cellular Monitoring

To evaluate the capability of the CHLM to monitor tissue and cellular processes, we decided to perform an angiogenesis assay using primary human umbilical vein endothelial cells (HUVECs). Cells were obtained C-12203 (from PromoCell, Heidelberg, Germany) and cultured using Endothelial Cell Growth Medium Ready-to-use C-22010 (from PromoCell, Heidelberg, Germany) following standard protocols. Additional reagents included DetachKit C-41200 (from PromoCell, Heidelberg, Germany), HEPES-buffered saline solution (BSS), Trypsin/EDTA (0.04%/0.03%), Trypsin Neutralizing Solution (TNS), and Trypan Blue for viability staining. Cells were cultured in Nunc EasyFlasks 75 (156472) (from Thermo Fisher Scientific, Alcobendas, Spain). The culturing protocol involved thawing, handling, and subculturing frozen cells. Once the cultures reached 70–90% confluency, they were used for experiments.

Two samples were prepared to be observed simultaneously: one with the CLHM, and another with a conventional inverted microscope. Sample preparation followed the angiogenesis assay protocol provided by IBIDI (AN19). Briefly, Matrigel^®^ Growth Factor Reduced, Phenol Red-Free, #356231 (from Corning Optical Communications S.L.U., Las Rozas de Madrid, Spain) was thawed overnight at 4 °C and added to μ-Slide 15 Well 3D plates 81506 (from IBIDI, catalog no. 81506; distributed by Inycom, Zaragoza). One well was left uncoated as a negative control to confirm that angiogenesis does not occur without a gel matrix. The two plates were placed in a humidified Petri dish and incubated for 60 min at 37 °C to allow Matrigel polymerization.

For cell detachment and resuspension, all reagents were prewarmed before starting. First, the medium was removed and discarded. Then, the flask was washed with HEPES BSS, after a few seconds it is removed. Trypsin/EDTA solution was added to the flask and left at room temperature for 5 min maximum. During this time, the flask was observed under the microscope until the cell layer was detached. TNS was added in equal parts to stop enzymatic activity. The suspension was collected, placed into a 15 mL tube, and centrifuged for 3 min at 220× *g*. The cells were resuspended in 1mL growth medium. After this step, viable cells were counted and 50 μL of HUVECs and EC medium mix was added to each of the wells (both Matrigel-coated and uncoated).

**Figure 5:**
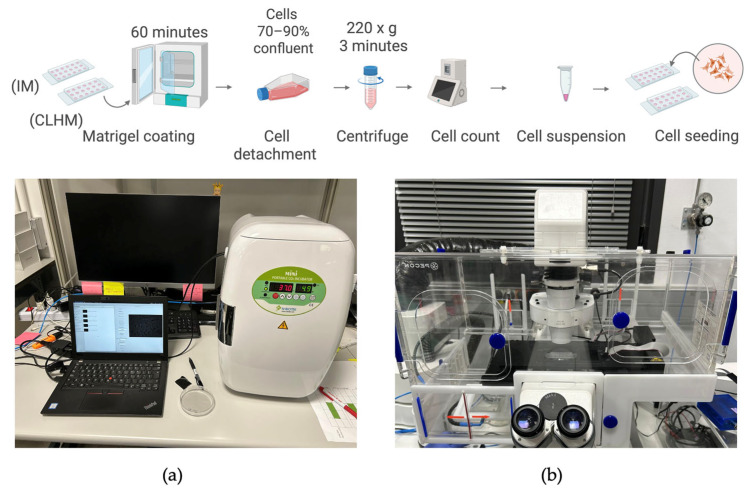
Sample preparation and setup. (**a**) CLHM setup inside CO_2_ incubator. (**b**) Inverted microscope setup.

Imaging was performed using both the CLHM and an inverted microscope (10× objective) over a 24 h period, sufficient to monitor cell morphology and tube formation dynamics. Figure 5 summarizes the protocol followed for sample preparation and imaging with both systems.

## 3. Results

### 3.1. Resolution Analysis

To evaluate the resolution of the CLHM, we initially performed a contrast-based analysis using intensity profiles extracted from reconstructed holograms acquired at five sample-to-camera distances (P1–P5). For each position, we selected a region of interest (ROI) in Group 7, Element 6 of the USAF 1951 resolution target, focusing on the horizontal line pattern. We then drew twenty lines perpendicular to the pattern and extracted the corresponding intensity profiles. To provide a more comprehensive analysis, we extended this procedure to additional elements within the same group (Elements 1 and 2) in order to construct the system’s Modulation Transfer Function (MTF). This allowed us to evaluate contrast across multiple spatial frequencies.

**Figure 6:**
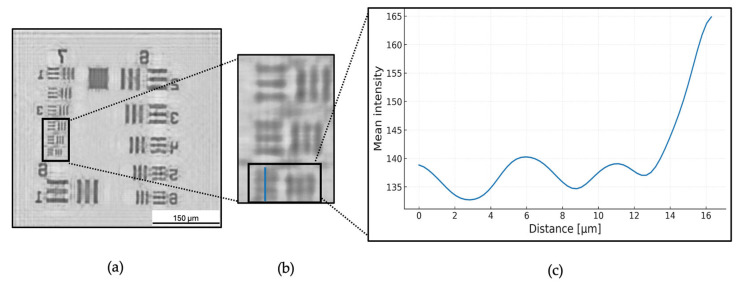
(**a**) P1 image after normalization, backpropagation and twin-image removal. (**b**) the region of interest (ROI) group 7, element 6 profile lines. (**c**) Mean intensity profile of P1.

Figure 6 illustrates the workflow for position P1 (11 mm) as a representative example. First, the hologram underwent processing that included normalization, twin-image removal, and numerical backpropagation (Figure 6a). Next, we identified the regions of interest and extracted twenty intensity profiles (Figure 6b). Although only a single line is depicted for clarity, twenty parallel lines were determined, and their profiles were obtained. Finally, we computed and plotted the average of these twenty profiles (illustrated in Figure 6c). The curve is Gaussian-smoothed for visualization only; all quantitative calculations used the raw data.

To quantify the resolution, the Michelson contrast formula was calculated for each intensity profile (Equation (1)):

(1) $$C_{M\;}=\;\frac{L_{\max}\;-L_{\min}}{L_{\max}\;+\;L_{\min}}$$

where $L_{max}$ corresponds to the maximum intensity (brightest region) and $L_{min}$ to the minumum intensity (darkest region). This calculation was applied to each individual profile across all elements. The resulting contrast values were then averaged for each element, and the mean Michelson contrast was plotted against the corresponding spatial frequency to generate the MTF.

**Figure 7:**
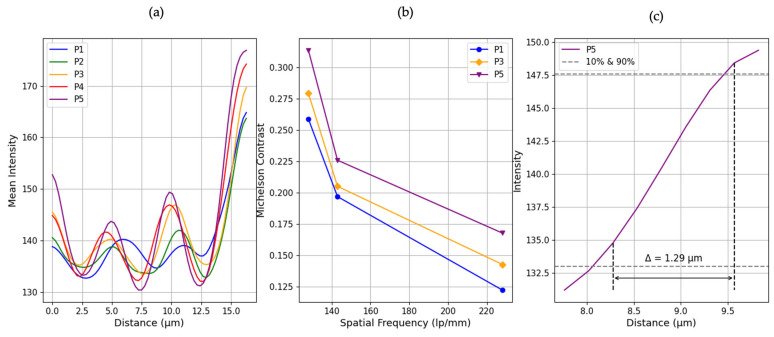
(**a**) Gaussian-smoothed mean intensity profiles extracted from horizontal line patterns in Group 7, Element 6 of the USAF 1951 target, for each sensor position (P1–P5). (**b**) Modulation Transfer Function (MTF) curves of P1, P3 and P5 computed from the Michelson contrast of averaged profiles across multiple elements (1, 2, and 6) of Group 7. (**c**) Estimated PSF for P5.

Figure 7a presents the mean vertical intensity cross-sections through the USAF 1951 target’s finest line pattern (Group 7, Element 6) at five sensor positions (P1–P5, corresponding to object–sensor distances of 11, 10, 9, 8 and 7 mm, respectively). This element was selected for visualization as it represents the highest spatial frequency tested (228 lp/mm), and therefore provides the most critical example of the system’s resolution limit. Each profile exhibits a repeating sequence of peaks and valleys that map the target’s transparent and opaque features, respectively. A clear trend emerges whereby bringing the sensor closer produces stronger intensity modulation. At the shortest distance (P5, 7 mm), the profiles show well-separated, pronounced peaks and deep troughs (valleys nearly dropping to the background level), indicating that the dark lines and bright spaces are sharply resolved. In contrast, at the largest distance (P1, 11 mm), the intensity variations are much less visible, peaks are reduced and valleys are elevated, reflecting optical diffraction that washes out fine detail and diminishes the distinction between line and space.

Figure 7b quantifies the MTF curves derived from the Michelson contrast of averaged intensity profiles from elements 1, 2, and 6 within Group 7. Each of these elements corresponds to a distinct spatial frequency: 128 lp/mm, 143 lp/mm, and 228 lp/mm, respectively. The resulting MTF curves reveal a trend, shorter sensor-to-sample distances consistently yield higher contrast. For instance, at 228 lp/mm (Element 6), the Michelson contrast increases from 0.122 at P1 to 0.167 at P5. This increase confirms enhanced resolution performance at closer distances. To obtain the system’s resolution estimate, we took the P5 intensity profile and detected the 10% and 90% of the curve at 133.00 intensity around 8.28 µm and 147.57 intensity near 9.57 µm. The distance between these points, known as the PSF width, was found to be 1.29 μm.

### 3.2. Biological Samples Analysis

**Figure 8:**
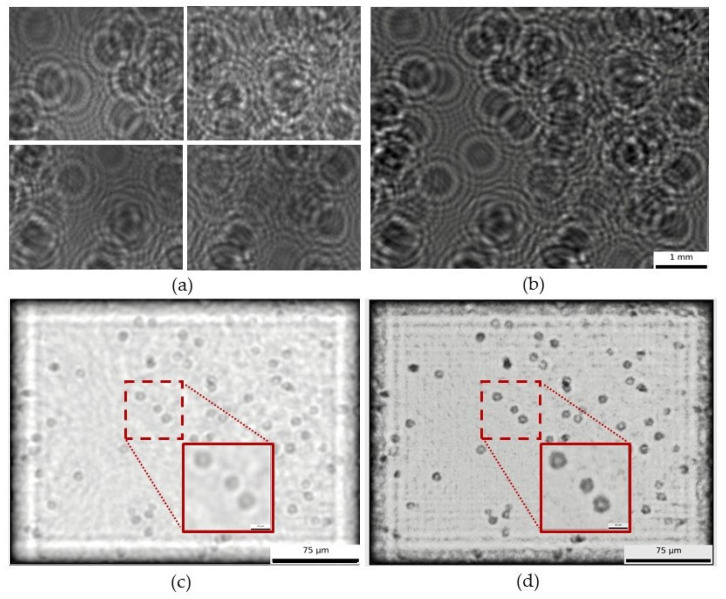
Raw images recorded by illuminating with a 2 × 2 LED grid (**a**), stitched mosaic (**b**), image reconstruction obtained by using only backpropagation (**c**), and reconstruction obtained by using both backpropagation and twin-image correction (**d**).

As a first demonstration of the ability of our CHLM for individual cellular monitoring, Figure 8 shows the image processing steps used to obtain the final view of yeast cells (*Saccharomyces cerevisiae*) from a wine sample in fermentation. The reconstruction process is illustrated using two approaches: backpropagation only versus backpropagation with twin-image removal. Although both processes allow cell resolution, the latter provides significantly clearer image and reduced distortion, making individual yeast cells more distinguishable. After reconstructing the image with our backpropagation and twin-image removal algorithms, we estimated cell concentration by applying a circular Hough transform (integrated into the analysis software), yielding 7.17 × 10^6^ cells/mL. The detection method was previously validated in detail by Benito-Altamirano et al. [2], showing a strong linear correlation with manual optical microscopy counts across 0.5 to 50 million cells/mL. Therefore, we did not repeat the validation here. In our study, we confirmed within the 0.5 to 50 million cells/mL range, supporting the method’s reliability.

**Figure 9:**
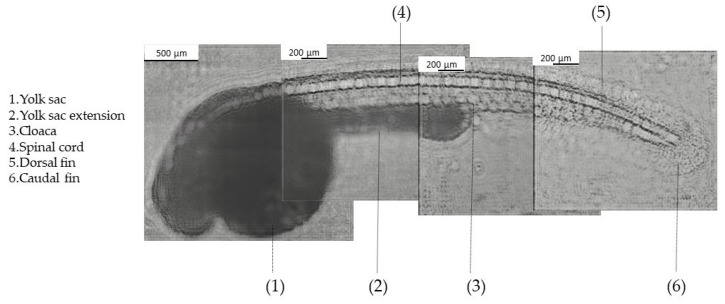
Composite image of zebrafish 72 hpf.

The second biological application involved imaging zebrafish embryos. To assess the CLHM’s ability to visualize small model organisms, a composite image was created from four sequentially captured segments of a 72 hpf zebrafish embryo (Figure 9). Each of the segments displays different anatomical features. In the anterior region, features such as the eye, otolith, heart, and liver were not clearly visible due to the high density of the yolk sac and its extension, which attenuate light penetration and limit the CMOS sensor’s ability to detect finer internal structures. While this limitation may restrict access to certain features, it does not significantly limit the system’s utility for a wide range of developmental biology studies. Other anatomical features are visible in the rest of the body, including the yolk sac, yolk sac extension, cloaca, spinal cord, dorsal and caudal fins. These structures are highly relevant in research involving zebrafish development, organogenesis, or toxicological analysis. Studies such as those by Sant et al. [38] and Tian et al. [39] have specifically examined features like the yolk sac and cloacal development, underscoring their importance.

**Figure 10:**
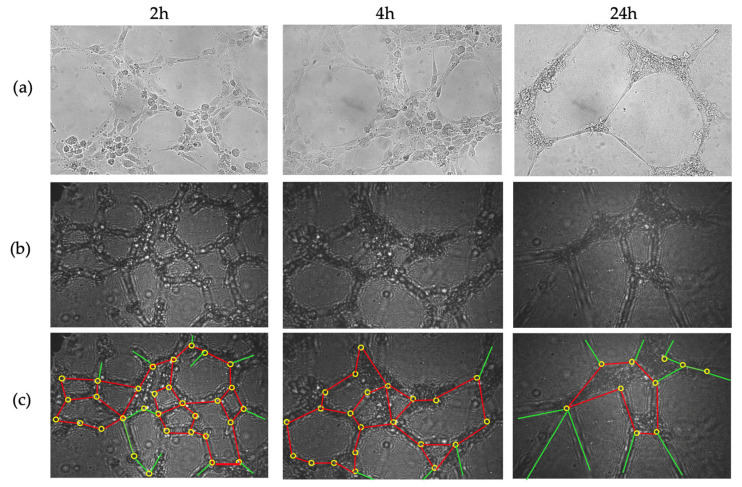
(**a**) Angiogenesis images taken with inverted microscope (after 2 h, 4 h and 24 h at left, middle and right, respectively), (**b**) Images without backpropagation, and (**c**) Images taken with CLHM without backpropagation with closed loops in red, non-looped branches in green and junctions in yellow. The images (**a**) are of 333 × 232 μm and (**b**) are 464 × 307 μm.

As a third approach, to monitor cellular dynamics and tissue evolution, Figure 10 shows the time-lapse progression of angiogenesis at 2, 4, and 24 h, captured using both the inverted microscope (a) and the CLHM (b and c). Both sets of images illustrate how HUVEC cells form tubular networks and extend branches over time, with the tubes gradually thinning as they reach their maximum extension. In angiogenesis assays, quantitative parameters such as the number of closed loops, and junctions, are typically measured. Here, we have overlaid these features on the raw images: closed loops in red (2 h: 9, 4 h: 6, 24 h: 1), junctions (branch intersections) in yellow (2 h: 31, 4 h: 22, and 24 h: 10), and non-loop branches (branches that do not form part of a loop) in green (Figure 10c). Table 1 includes the values of the raw counts of these features. These absolute counts highlight a clear decreasing trend in all features over time, likely reflecting progressive thinning of the vascular network and the disruption of branch connections.

The annotations were manually performed based on visual inspection of the raw holograms, following a similar methodology to conventional microscopy. Since no automated detection algorithms were used, the potential for false detection is equivalent to typical manual counting practices in standard angiogenesis assays. This approach is commonly accepted in biological imaging and provides sufficient reliability for observing comparative trends over time.

**Figure 11:**
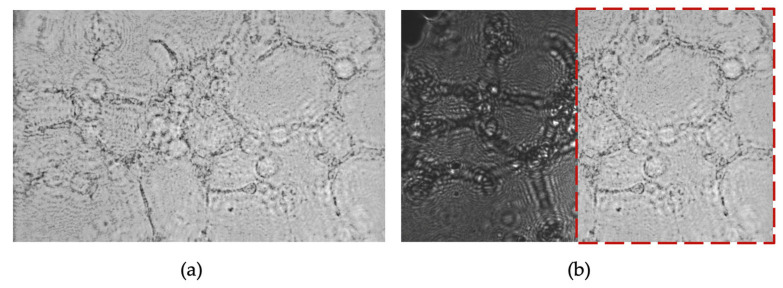
(**a**) Backpropagated and twin-image-removed 2 h time point (z1 = 1800 μm), and (**b**) Overlap of (**a**) on the raw image. The images (**a**,**b**) are 464 × 307 μm.

To minimize post-processing time, we omitted digital backpropagation and twin-image removal for these images because the raw holograms already allowed clear identification of some angiogenic features. For illustration, Figure 11 shows the 2 h time point after applying both procedures. While some cells are sharply reconstructed, others remain blurred due to the uneven Matrigel layer, which places cells at different focal depths. In CLHM, multi-height reconstructions can be performed to refocus each region, allowing users to choose the plane that best visualizes the structures of interest.

## 4. Discussion

In this study, we demonstrated that the CLHM (Figure 2) can achieve a resolution better than 2.19 µm at a working distance of 7 mm (position P5 in Figure 7). The estimated resolution value was determined using the P5’s curve resulting in 1.29 µm (Figure 7c). This resolution compares favorably with some miniature microscopes [1,3,4,6], smartphone based [11], similar on-chip microscopes [13,15,16] which reported resolutions in the 3–5.52 μm range. This high resolution combined with the device’s compact geometry (6 × 6 × 4.5 cm^3^), enables the visualization of biological structures such as tissues, individual cells, and small organisms. The short working distance facilitates a miniaturized and scalable imaging platform, well-suited for point-of-care (PoC) diagnostics, real-time in vivo and in situ biological monitoring, and drug screening applications.

Image reconstruction in the CLHM system is performed post-acquisition and includes normalization, image stitching, backpropagation, and twin-image removal. The processing time varies depending on the image resolution. For instance, with a conventional multi-field acquisition (3 × 3 grid with a 3072 × 2048 px resolution and the camera at 60 fps) it takes approximately 31.46 s to perform the normalization, stitching, backpropagation and twin image on a standard workstation. Additionally, the imaging acquisition times with the same configuration of grid and resolution requires approximately 166.7 ms per image. Although the reconstructed-phase images are not immediately available and need several seconds to be generated, the raw holographic images can already provide valuable qualitative feedback during the experiments. For example, researchers can observe particles flowing, sample movement, or early signs of tissue dynamics directly from the raw data, enabling in situ monitoring without disturbing or removing the sample from the incubator. Future work will focus on optimizing the reconstruction pipeline to reduce processing time and support near real-time post-processing for live-feedback applications.

In the context of wine fermentation, the CLHM successfully resolves and identifies yeast cells which are key parameters for monitoring and controlling fermentation quality. Although the present analysis was conducted in a static chamber rather than a flowing setup, it establishes the system’s technical feasibility and lays the ground for flowing microfluidic implementations directly connected to fermentation tanks. Such a setup would enable real time in situ analysis of yeast dynamics under continuous flow. Furthermore, this adaptability paves the way for broader applications in flow-based biological systems, such as suspension of cell cultures for disease modeling and therapeutic screening.

Regarding zebrafish imaging, we successfully visualized multiple anatomical structures, including the yolk sac, cloaca, spinal cord, and dorsal and caudal fins. Observing these structures will enable us to model several disorders. The spinal cord can be used to model musculoskeletal and fin development disorders such as congenital scoliosis, muscular dystrophy, spinal cord injury models and motor neuron disorders. In the case of the dorsal and caudal fins, some of the models that can be studied include malformations, fin regeneration, and musculoskeletal abnormalities. Moreover, the yolk sac and its extension might allow us to study nutrient absorption, lipid metabolism, and starvation effects. Lastly, the cloaca can allow for gut motility sides and excretion defects. For now, anatomical structures such as the eye, liver, heart, and otoliths cannot be resolved, likely due to light attenuation in dense tissues, limiting the number of applications it can be used for. These limitations may be addressed in the future through improved illumination, enhanced contrast, or the incorporation of fluorescence imaging, which is currently under development.

The CLHM exhibited strong performance in monitoring angiogenesis tube formation assays, displaying key morphological features such as closed loops, junctions, and non-looped branches directly in the raw images (Figure 10). These features were easily identifiable without the need for any processing after acquisition. Since the essential tubular structures and connections were already visible, we avoided additional image enhancement or extensive post-processing, which typically requires significant computational effort. Figure 11 demonstrates the effect of applying digital backpropagation to an image taken at the 2 h time point. While backpropagation can sharpen the focus of cells located at the optimal reconstruction depth, it tends to blur other cells when the sample spans multiple depth planes. In our assay, the Matrigel matrix was uneven, resulting in endothelial cells residing at various focal depths. Instead of conducting multi-height reconstructions for each region, we chose to bypass this computational step.

As an initial step, we quantified the absolute number of morphological features such as loops and junctions using raw images. While these values are useful to observe general trends, a comprehensive statistical analysis will require the acquisition and analysis of a large number of images. The wide field of view offered by our microscope will facilitate this in future studies. We consider this work a foundational step toward positioning the CLHM as a viable tool for angiogenesis assay monitoring.

In conclusion, the CLHM emerges as a compact and cost-effective platform that bridges the gap between advanced microscopy and accessible, real-time in situ biological monitoring. Its versatility, from microbial monitoring to vascular dynamics, demonstrates its potential to transform biological research, from single cells to whole organisms. Although currently limited to brightfield imaging, ongoing developments in fluorescence integration, resolution enhancement, and support for real-time imaging of flowing samples in microfluidic devices are expected to extend its capabilities for in situ and in vivo imaging. With these improvements, the CLHM has the potential to become a powerful tool not only in laboratory settings but also in decentralized diagnostics, environmental monitoring, and educational environments, redefining how and where high-resolution microscopy can be performed.

## Figures and Tables

**Table 1:** Quantification of angiogenesis parameters.

System	Time Point	Image Size (μm)	Closed Loops (Raw Count)	Junctions (Raw Count)
Inverted microscope	2 h	333 × 232	8	37
	4 h	333 × 232	6	26
	24 h	333 × 232	2	10
CLHM	2 h	464 × 307	9	31
	4 h	464 × 307	6	22
	24 h	464 × 307	1	10

## Data Availability

The original contributions presented are included in the article. Data and code are available from the corresponding author upon reasonable request. If required, access to the GitLab repository can be granted under specific conditions.
